# *BRCA1* Exon 11 Mutations in Breast Cancer: A Study From Pakistan

**DOI:** 10.1155/genr/5544418

**Published:** 2025-08-26

**Authors:** Murad Ali, Aziz Uddin, Sajid Ul Ghafoor, Atta Ur Rehman

**Affiliations:** ^1^ Department of Zoology, Hazara University, Mansehra, Khyber Pakhtunkhwa, Pakistan, hu.edu.pk; ^2^ Department of Internal Medicine and Medical Specialties (DIMI), University of Genoa, Genoa, Italy, unige.it; ^3^ Department of Biotechnology and Genetic Engineering, Hazara University, Mansehra, Khyber Pakhtunkhwa, Pakistan, hu.edu.pk

**Keywords:** *BRCA1*, breast cancer, genetic susceptibility, mutational screening, Pakistan

## Abstract

Breast cancer ranks among the top causes of cancer‐related deaths in women around the globe, with genetic mutations in the *BRCA1* gene being a frequent cause of breast or ovarian cancer. This study investigates hotspot mutations in exon 11 of the *BRCA1* gene among Pakistani women diagnosed with breast cancer. Thirty clinically diagnosed breast cancer patients, all women, were enrolled in the current study, and high‐quality DNA was extracted from peripheral blood samples. Two of the twenty‐five successfully sequenced samples had a homozygous missense variant (c.2312T > C: p.Leu771Ser) detected by Sanger sequencing after PCR amplification. Upon investigation in the ClinVar database, the identified variant showed conflicting interpretations of pathogenicity. Demographic data highlighted an early disease onset, showing that 56% of patients were under 50 years of age. The need for genetic screening was further supported by the fact that 24% of the patients had a positive family history of cancer. Our study emphasizes the necessity of screening *BRCA1* gene mutations to better understand the pathogenic potential of the identified variants in the Pakistani population.

## 1. Introduction

Breast cancer is clinically a heterogeneous disease presenting different subtypes, outcomes, and behaviors, of which the most typical symptoms include the formation of lumps, skin dimpling, tenderness, and redness [1]. Breast cancer encompasses several subtypes distinguished by where the cancer cells begin and how they behave. These include noninvasive forms such as ductal carcinoma in situ (DCIS); invasive types like ductal and lobular carcinomas; and less common types, including inflammatory breast cancer, triple‐negative breast cancer (TNBC), and Paget’s disease of the breast [2]. Both genetic and environmental components are involved in breast cancer development; however, environmental components, such as a fatty diet, smoking, depression, stress, delayed pregnancy, early menarche, and late menopause, are rather easier to manage than the racial/genetic components [3]. The use of exogenous hormones (e.g., contraceptives) and prolonged exposure to endogenous estrogen, such as with early menarche or late menopause, are also associated with increased breast cancer risk [4]. An earlier diagnosis of breast cancer has positive prospects (reducing morbidity or mortality) for the patients, but a delayed diagnosis results in tumor growth and metastasis, thus necessitating extensive treatment, mastectomy, and long‐term adjuvant therapy [5, 6]. In 2013, 1.8 million cases of breast cancer and 464,000 deaths were reported globally [7]. As per 2022 estimates, the worldwide prevalence of breast cancer gradually increased to approximately 2.3 million cases and 670,000 deaths, thus making breast cancer the most frequently diagnosed cancer among women in 157 out of 185 countries worldwide [8]. In 2020, Asia accounted for approximately 45% of all global breast cancer cases, representing a major regional healthcare burden and highlighting the need for targeted prevention and control strategies (World Economic Forum, 2023) [9]. Breast cancer, lung cancer, and oral cancer are among Pakistan’s most frequently reported cancer types. According to the National Cancer Registry of Pakistan, breast cancer accounts for 21.4% of all other cancers, making it the most common overall [10]. Furthermore, one in every nine women has a lifetime risk of being diagnosed with breast cancer [11].

Data from the International Agency for Research on Cancer (IARC) Global Cancer Observatory indicates that in 2020, breast cancer was the predominant cancer among Pakistani women, with an incidence of 34.2 cases and a mortality of 18.6 deaths per 100,000 women, highlighting its considerable health impact (IARC, 2020) [12]. In the same year, in a breast cancer awareness event in Lahore, the WHO highlighted that “Approximately one‐ninth of Pakistani women will face breast cancer at some stage in their lives” (World Health Organization, 2020) [13]. Inherited genetic alterations underlie roughly 5%–10% of breast cancer cases. Thus, there is a higher likelihood of the disease emerging at a younger age in hereditary cases than in noninherited instances [14]. Women carrying pathogenic or likely pathogenic (P/LP) variants in moderate‐ or high‐penetrance genes, such as *BRCA1, BRCA2, PALB2, TP53, CDH1, ATM, CHEK2, NF1, PTEN, BARD1, RAD51C, RAD51D*, and *STK11*, have an increased risk of developing hereditary breast cancer [15]. Inherited mutations in the tumor‐suppressor genes like *BRCA1* and *BRCA2* confer a hereditary predisposition to breast cancer [16]. Carriers of pathogenic *BRCA1* variants exhibit an estimated cumulative breast cancer risk of approximately 72% by the age of 80, while individuals harboring *BRCA2* mutations demonstrate a comparable lifetime risk of around 69% [17]. Germline alterations in *BRCA1* drive about 40%–45% of familial breast cancers but represent only 2%‐3% of all breast cancer cases, since *BRCA1* mutations are uncommon in sporadic tumors [18]. Individuals who inherit the *BRCA1* mutations confront a lifetime breast cancer risk of 65%–80% and an ovarian cancer risk of 37%–62%, whereas *BRCA2* mutation carriers face a 45%–85% chance of breast cancer and a 11%–23% chance of ovarian cancer over their lifetimes [19]. Moreover, the literature implies that women with *BRCA1* mutations have an average cumulative breast cancer risk of 65% by age 70, while those with *BRCA2* mutations have a corresponding risk of 45%, based on pooled data from 22 studies of individuals unselected for family history [20]. The tumor suppressor *BRCA1* is recognized as a key player in the DNA damage response. The protein encoded by *BRCA1* regulates transcription, apoptosis, HR‐mediated DNA repair, and cell cycle checkpoints [21]. Pathogenic *BRCA1/2* variants occur at an allele frequency of approximately 0.13%–0.25% (1 in 800–1 in 400) in the general population, rising to about 2.5% (1 in 40) among those of Ashkenazi Jewish ancestry. Reported cancer penetrance for these germline mutations is highly variable, and even individuals within the same family carrying an identical *BRCA1/2* alteration can exhibit markedly different risk profiles [22]. The *BRCA1* gene spans roughly 81 kb of genomic DNA and comprises 24 exons, 22 of which constitute the protein‐coding region. These exons give rise to a ∼7 kb mRNA transcript that is translated into a 1863–amino‐acid polypeptide [23]. Spanning 3426 base pairs, exon 11 of the *BRCA1* gene constitutes a substantial central exon, accounting for 60% of its protein‐coding region [24]. Splicing of exon 11 yields three principal *BRCA1* transcript variants: the full‐length isoform retaining all of exon 11, the D11 isoform omitting exon 11 entirely, and the D11q isoform incorporating only a 117–base‐pair segment of exon 11 [25]. *BRCA1* deficiency leads to abnormalities in multiple cell cycle checkpoints and centrosome duplication, while the resulting genetic instability activates DNA damage responses that inhibit proliferation and induce apoptosis, mechanisms that must be overcome for *BRCA1*‐mutant cells to progress into malignant tumors [26]. Our study aimed to uncover disease‐causing variants in *BRCA1 among* female breast cancer patients belonging to the Abbottabad district, Pakistan.

## 2. Materials and Methods

### 2.1. Ethical Approval and Patient Enrollment

Following approval of our study by the Ethics Review Committee of Hazara University, Mansehra, and permission from the hospital authorities, informed consent was obtained from all participants or their legal guardians before collecting blood samples. Patients diagnosed with breast cancer and subsequently enrolled at the Institute of Nuclear Medicine, Oncology and Radiotherapy (INOR), Abbottabad, were selected for this study between September 6, 2021, and October 7, 2021. During this period, a total of 30 clinically diagnosed breast cancer patients were included in the study, and blood samples were collected accordingly. Blood samples were immediately transferred to the Molecular Genetics Research Laboratory, Department of Biotechnology and Genetic Engineering (BGE), Hazara University Mansehra, Pakistan, and frozen at −30°C until further use. Demographic and clinical data of breast cancer patients were recorded on a predesigned questionnaire. Only the patients’ clinical and demographic data were recorded, which included information such as age at menarche, age at diagnosis, number of children, weight, disease stage, comorbidities, treatment, and self‐reported family history of breast cancer. Although detailed family pedigrees were not collected, patients were asked verbally to report the presence or absence of breast cancer in their family history.

### 2.2. DNA Extraction

The phenol–chloroform–isoamyl alcohol (PCI) DNA extraction method was used to extract genomic DNA from the peripheral blood samples. After taking the blood samples out of the deep freezer (−30°C), 300 μL of blood was taken from each EDTA tube using a calibrated pipette (range: 20–300 μL) and transferred into 1.5 mL Eppendorf tubes containing 303 μL of the prepared lysis stock (lysis buffer, 2‐mercaptoethanol, and proteinase‐K) to lyse the blood cells. Blood samples were mixed gently with the lysis stock. After that, the samples were incubated at 55°C–60°C for one hour, and 300 μL volume of PCI (25:24:1) was added to the lysed sample, and thoroughly vortexed or shaken by hand for approximately 20 s. PCI denatures and eliminates cellular proteins, allowing genomic DNA to separate into the aqueous phase following a 20‐minute centrifugation at 8000 RPM (≈7197 × g) (Thermo Fisher Scientific, Waltham, MA, USA). The aqueous upper layer was carefully taken through a pipette and transferred into a new Eppendorf tube. 500 μL of isopropanol was added to each Eppendorf tube containing the clear supernatant and mixed by vortexing. At −30°C, the samples were incubated overnight. After overnight incubation, the samples were centrifuged at 8000 RPM (≈7197 × g) for 20 min using a Thermo Fisher Scientific Heraeus Pico 21R centrifuge (Thermo Fisher Scientific, Waltham, MA, USA) to pellet the DNA. The supernatant was carefully discarded. To purify the DNA, 500 μL of 70% ethanol was added to each Eppendorf tube containing the DNA pellet. The samples were centrifuged at 8000 RPM (≈7197 × g) for 5 min. After discarding the ethanol, the samples were incubated at 50°C for around 20 min in a dry‐bath incubator. Finally, the DNA pellet was dissolved by vortexing each sample with 50 μL of sterile water, and the purity and yield of the isolated DNA were assessed using a NanoDrop spectrophotometer (Thermo Fisher Scientific). Samples with a 260/280 ratio between 1.8 and 2.0 were marked suitable for downstream applications. To further verify DNA integrity, samples were electrophoresed through a 0.8% agarose gel in 1 × TAE buffer supplemented with 0.5 μg/mL ethidium bromide at 100 V for 30 min, and DNA quality was assessed by evaluating band sharpness and absence of smearing [27].

### 2.3. Primer Design, PCR Amplification, and Sanger Sequencing

**Figure 1:**
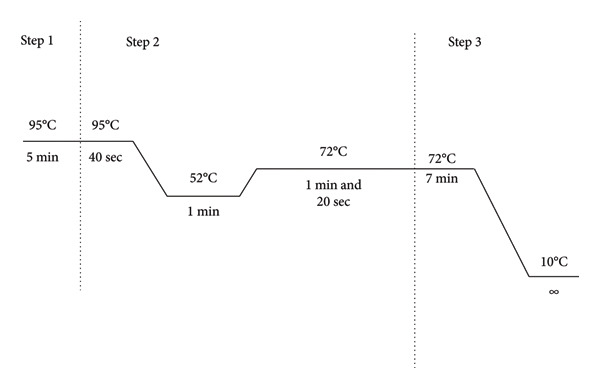
PCR amplification conditions.

A pair of primers targeting *BRCA1* exon 11 (Forward 5′‐CTG​AGG​AGG​AAG​TCT​TCT​ACC‐3′; Reverse 5′‐GCA​TCA​AGT​TCA​CTT​TCT​TCC‐3′) was used to amplify a 731‐bp fragment. PCR reactions (30 μL) were assembled with 14 μL nuclease‐free water, 10 μL Axen Taq PCR Master Mix (2 ×; Macrogen, Seoul, Republic of Korea, Cat. No. MG‐E‐004‐5) containing 1 × PCR buffer, MgCl_2_, dNTPs, and Taq DNA polymerase, 2 μL of each primer (working stock at 30 pmol/μL), and 2 μL extracted genomic DNA. Thermal cycling was performed on Applied Biosystems GeneAmp PCR System 9700 thermal cycler: 95°C for 5 min; 35 cycles of 95°C for 40 s, 52°C for 1 min, and 72°C for 1 min and 20 s; and a final extension at 72°C for 7 min (Figure 1). PCR products were resolved on a 1% agarose gel against a 100 bp plus ladder (Promega BenchTop) to confirm size and yield. Amplicons were purified and submitted for Sanger sequencing at Macrogen Inc. (Seoul, Korea) on an Applied Biosystems 3730xl DNA Analyzer. Chromatograms were inspected in UGENE (v1.16.1) and aligned against the *BRCA1* reference sequence (ENSG00000012048) for variant calling.

## 3. Results

### 3.1. Patients’ Demographic Information

A total of 30 women diagnosed with breast cancer were enrolled in our study; however, five patients were later excluded from the final analysis due to incomplete clinical and demographic data. The participants’ ages ranged from 31 to 72 years, with a mean ± SD age at diagnosis of 48 ± 12.7 years. Most patients were ≤ 50 years old (Table 1). All patients were married, with an average of 3.36 ± 1.57 children, and a mean menarche age of 11.68 years (range: 11–13 years). The mean age at marriage was 19.7 years (range: 16–23 years), while the mean age at first delivery was 21 years (range: 17–25 years). All patients had children and breastfed their babies for an average of 1 year. Only 36% of the patients had hypertension, 16% had diabetes, 4% had hepatitis, and the rest had no reported comorbidity. A positive family history of cancer (breast or other cancers) was reported by 24% of the study participants. Of the 25 patients, five were diagnosed with stage IV breast cancer, the others with stages I to III, and only one case was identified as TNBC. Out of 25 patients, 16 received chemotherapy and 9 underwent surgical treatment.

**Table 1:** Patient demographics and clinical data (*n* = 25).

Sample ID	Age at menarche	Age at BC diagnosis	Number of children	Weight (kg)	Stage	Chronic diseases	Treatment	Family history (BC)
M1	12	35	5	58	3	None	Chemotherapy	No
M2	11	53	1	55	4	None	Chemotherapy	No
M3	13	68	2	75	2	Diabetes	Chemotherapy	No
M4	13	39	5	55	2	None	Chemotherapy	No
M5	11	63	5	60	3	Hypertension	Surgery	No
M6	12	32	1	60	1	None	Surgery	No
M7	13	58	3	80	DCIS	Hypertension	Surgery	No
M8	11	55	5	75	4	Diabetes, hypertension	Chemotherapy	Yes
M9	12	48	5	63	2	None	Chemotherapy	No
M10	11	31	3	60	1	None	Surgery	Yes
M11	11	60	5	70	3	Hypertension	Chemotherapy	No
M12	12	43	4	60	4	None	Chemotherapy	Yes
M13	11	39	2	63	2	None	Chemotherapy	No
M14	11	51	6	72	DCIS	Hypertension	Surgery	No
M15	13	46	3	65	3	Hypertension	Chemotherapy	Yes
M16	11	72	3	53	4	None	Chemotherapy	No
M17	12	36	1	65	2	None	Surgery	No
M18	11	52	3	75	2 (TNBC)	Diabetes, hypertension	Chemotherapy	Yes
M19	11	36	2	50	3	None	Chemotherapy	No
M20	11	42	1	57	3	None	Chemotherapy	No
M21	13	36	4	60	2	None	Chemotherapy	No
M22	12	56	2	65	4	Hypertension	Chemotherapy	No
M23	11	31	5	60	2	None	Surgery	Yes
M24	12	72	3	80	DCIS	Diabetes, hypertension	Surgery	No
M25	11	46	5	58	2	Hepatitis	Surgery	No

Abbreviations: DCIS, ductal carcinoma in situ; TNBC, triple‐negative breast cancer*.*

### 3.2. Qualitative Assessment of DNA

**Figure 2:**
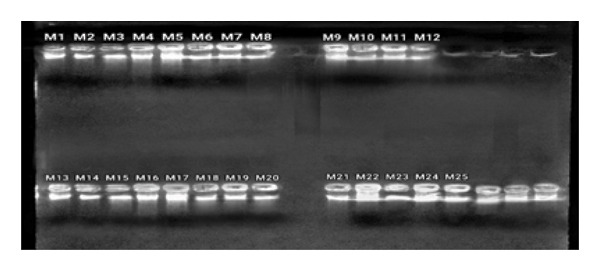
DNA extraction results (M1–M25).

All 25 extracted blood samples yielded good‐quality DNA (Figure 2).

### 3.3. Amplification of Exon 11 of *BRCA1*

**Figure 3:**
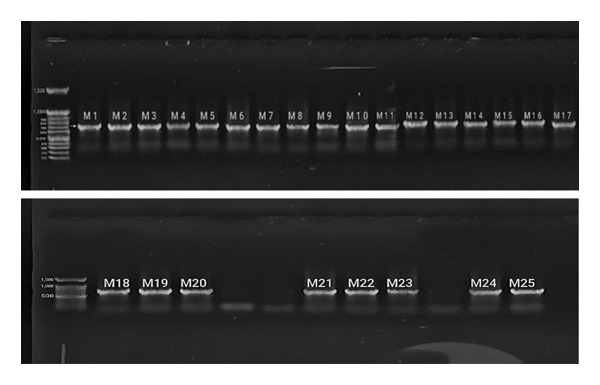
PCR‐amplified 731‐bp region (M1–M25).

PCR amplification of *BRCA1* exon 11 was performed using a specific primer pair, yielding 731‐bp fragments (Figure 3).

### 3.4. Sequence Analysis

Amplified PCR products were submitted to Macrogen Inc. (Seoul, Republic of Korea) for unidirectional Sanger sequencing using the forward primer. UGENE (ver. 1.16.1) was used to visualize the chromatograms and DNA sequence. The resulting sequences were aligned using the Ensembl reference genome (Accession No: ENSG00000012048) [28].

**Figure 4:**
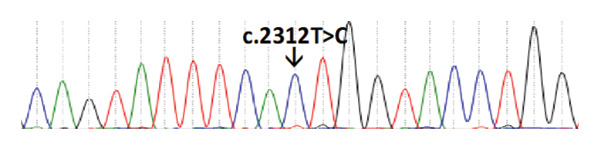
Chromatogram showing a T > C change in *BRCA1*.

We detected a homozygous missense variant (c.2312T > C:p.Leu771Ser) in *BRCA1* exon 11 among two unrelated patients, M13 and M21 (Figure 4).

Patient M13 was 39 years old and weighed 63 kg, while patient M21 was 36 years old and weighed 60 kg. Both patients were diagnosed at stage II, with no other chronic illnesses, under treatment with chemotherapy, and had no family history of breast cancer or other cancers. Despite the presence of this homozygous variant in these two unrelated individuals with similar clinical profiles, the c.2312T > C alteration remains classified as a variant of uncertain significance (VUS) in ClinVar, as current evidence is insufficient to support its reclassification.

### 3.5. *In Silico* Analysis

The identified variant was subjected to a thorough in silico analysis using several bioinformatics tools and databases (Table 2). By ACMG guidelines, the Variant Validator and the Ensembl Genome Browser (GRCh37) were used for the genomic nomenclature of the identified variant. The minor allele frequency (MAF) was calculated using the Genome Aggregation Database (gnomAD v2.1.1) with reference to the South Asian population, while ClinVar (accessed in 2022) and HGMD Professional (2022 version) were checked to confirm the variant’s previously reported status. Several online tools were consulted to assess the potential pathogenicity of the mutations, including PolyPhen‐2, Mutation Assessor, Mutation Taster, LRT, SIFT, PROVEAN, REVEL, CADD, and DANN. Finally, species‐wise conservation of the mutations was scored using GERP and PhyloP100 (Table 2). The c.2312T > C change was classified as a VUS by ACMG and ClinVar, probably damaging by PolyPhen‐2, and damaging by SIFT and PROVEAN. However, LRT and REVEL classified the allele as neutral and benign, respectively. A CADD‐PHRED score of 22.7 indicates a possibly damaging effect of the c.2312T > C change on the protein (Table 2).

**Table 2:** Genomic features and in silico analysis of the identified nucleotide variations.

Gene symbol	*BRCA1*
Ensembl Gene ID	ENSG00000012048
Transcript	IDNM_007294.4
Protein ID	NP_009225.1
Genomic change	Chr17:41245236:A > G
cDNA change	c.2312T > C
Protein change	p.(Leu771Ser)
gnomAD MAF	0.000003983
ACMG	VUS
ClinVar	VUS
HGMD	Reported
PolyPhen‐2	Probably damaging
Mutation Taster	Polymorphism
Mutation Assessor	Medium
LRT	Neutral
SIFT	Damaging
PROVEAN	Damaging
REVEL	Benign
CADD Score	22.7
DANN Score	0.9976
GERP Score	5.1399
PhyloP100 Score	1.873

*Note:* ClinVar (Clinical Variation Database), PolyPhen‐2 (Polymorphism Phenotyping v2), PROVEAN (Protein Variation Effect Analyzer), CADD Score (Combined Annotation Dependent Depletion Score), DANN Score (Deleterious Annotation of Genetic Variants Using Neural Networks), GERP Score (Genomic Evolutionary Rate Profiling Score), PhyloP100 Score (Phylogenetic *P*‐value for 100 Vertebrates).

Abbreviations: ACMG, American College of Medical Genetics; HGMD, Human Gene Mutation Database; LRT, likelihood ratio test; REVEL, Rare Exome Variant Ensemble Learner; SIFT, Sorting Intolerant From Tolerant; VUS, variant of uncertain significance.

## 4. Discussion

This study identified a homozygous missense variant (c.2312T > C:p.Leu771Ser) in the *BRCA1* gene among breast cancer patients admitted at the INOR Cancer Hospital in Abbottabad, Pakistan. The appearance of the p.Leu771Ser mutation in a homozygous state in both patients likely reflects the prevalence of endogamy or parental consanguinity in the region, although a recent study suggested a declining trend of the latter in Northwest Pakistan [29]. Further, whether the two patients carrying the same allele have a shared ancestry is unclear. Nevertheless, similar studies conducted in the region have documented numerous founder alleles causing diverse monogenic conditions in the region [30, 31].

To the best of our knowledge, this mutation has not previously been reported in the Pakistani population. Although its clinical significance remains uncertain, the novelty of the identified variant in our cohort suggests that this may be a population‐specific mutation that could possibly contribute to hereditary breast cancer risk.

This variant was classified as a VUS by ACMG and ClinVar but predicted as damaging by PolyPhen‐2, SIFT, and PROVEAN. *BRCA1* and *BRCA2* germline mutations are linked to a high percentage of hereditary breast and ovarian cancers. The prevalence of breast and ovarian cancer in high‐risk families owing to mutations in tumor suppressor genes varies among populations [32]. *BRCA1* and *BRCA2* (*BRCA1/2*) are the most commonly studied genes in Caucasian cancer patients, although there are few studies on Asians. Muhammad et al. discovered *BRCA1/2* variants in pancreatic cancer patients, suggesting the greater significance of *BRCA* mutations beyond breast cancer [33], whereas Rashid et al. observed recurrent and founder *BRCA1* alterations among families with breast and ovarian cancer [34]. Ahmad et al. used whole‐exome sequencing in the Pashtun population and identified several new mutations in exons 9 and 11 of the *BRCA1* gene, indicating variations in mutation patterns by area and ethnicity [35]. Strong correlations between *BRCA1* SNPs (such as rs8176237, rs1060915, and rs799912) and both benign and malignant breast cancers in Pakistani women have been reported [36]. Our results show a distinct missense mutation not yet seen in national databases, which sets them apart from the general trend of significant *BRCA* contribution to breast cancer in Pakistan. This highlights the need to broaden genetic screening initiatives to encompass rare mutations and underrepresented populations. Furthermore, constitutional *BRCA1* promoter methylation is also strongly linked to TNBC [37], according to methylation studies like those conducted by Muhammad et al., indicating that both genetic and epigenetic abnormalities contribute to cancer in Pakistani patients [38]. Notwithstanding the importance of our findings, several limitations need to be noted. The applicability of the findings may be restricted by the limited sample size and the unavailability of extended family history data. Furthermore, we only looked at exon 11, ruling out any possible significant mutations in other exons. The uncertain classification of the p.Leu771Ser variant calls for more research using functional assays, family cosegregation evaluation, and large‐scale studies.

Future studies should consider integrative analysis of both genetic and epigenetic data, as well as whole‐genome or exome sequencing across various Pakistani ethnicities. To ascertain the therapeutic value of novel or rare variants like p.Leu771Ser, larger cohorts and thorough phenotypic correlations would be necessary. In Pakistani populations, confirming its pathogenicity would have a significant impact on risk assessment, genetic counseling, and personalized cancer prevention strategies.

## 5. Conclusion

*BRCA1* gene mutations vary based on genetic background, environmental factors, and family history of cancer. Here, we identified a homozygous missense variant (c.2312T > C:p.Leu771Ser) in *BRCA1* as the likely cause of breast cancer among selected patients, reinforcing the need to integrate genetic screening into clinical care for high‐risk populations. ACMG and ClinVar reported the mutation as a VUS; however, in silico tools predicted a potentially damaging effect. Demographic data revealed that over half the patients were diagnosed before age 50, indicating early disease onset. These findings reflect the global diversity in *BRCA1* mutations. Functional validation of variants and comprehensive studies are essential, given the high prevalence of breast cancer in Pakistan. We recommend integrating genetic screening into clinical settings for early diagnosis and treatment strategies for breast cancer.

## Conflicts of Interest

The authors declare no conflicts of interest.

## Funding

This research did not receive any specific grant from funding agencies in the public, commercial, or not‐for‐profit sectors.

## Data Availability

The datasets generated and/or analyzed during the current study are available from the corresponding author upon reasonable request.

## References

[1] Orrantia-Borunda E. , Anchondo-Nuñez P. , Acuña-Aguilar L. E. , Gómez-Valles F. O. , and Ramírez-Valdespino C. A. , Subtypes of Breast Cancer, Breast Cancer. (2022) 31–42, 10.36255/exon-publications-breast-cancer-subtypes. 36122153

[2] American Cancer Society , Types of Breast Cancer, 2021, https://www.cancer.org/cancer/breast-cancer/about/types-of-breast-cancer.html.

[3] Rudolph A. , Chang-Claude J. , and Schmidt M. K. , Gene–Environment Interaction and Risk of Breast Cancer, British Journal of Cancer. (2016) 114, no. 2, 125–133, 10.1038/bjc.2015.439, 2-s2.0-84955176894. 26757262 PMC4815812

[4] Chen W. Y. , Exogenous and Endogenous Hormones and Breast Cancer, Best Practice & Research Clinical Endocrinology & Metabolism. (2008) 22, no. 4, 573–585, 10.1016/j.beem.2008.08.001, 2-s2.0-54849426520. 18971119 PMC2599924

[5] Unger-Saldaña K. and Infante-Castañeda C. , Delay of Medical Care for Symptomatic Breast Cancer: A Literature Review, Salud Publica de Mexico. (2009) 51, s270–s285, 10.1590/s0036-36342009000800018, 2-s2.0-70349635764. 19967283

[6] Ho P. J. , Cook A. R. , Binte Mohamed Ri N. K. , Liu J. , Li J. , and Hartman M. , Impact of Delayed Treatment in Women Diagnosed With Breast Cancer: A Population-Based Study, Cancer Medicine. (2020) 9, no. 7, 2435–2444, 10.1002/cam4.2830. 32053293 PMC7131859

[7] Fitzmaurice C. , Dicker D. , Pain A. et al., The Global Burden of Cancer 2013, JAMA Oncology. (2015) 1, no. 4, 505–527, 10.1001/jamaoncol.2015.0735, 2-s2.0-84994730525. 26181261 PMC4500822

[8] World Health Organization , Breast Cancer: Key Facts and Global Impact, 2024, https://www.who.int/news-room/fact-sheets/detail/breast-cancer.

[9] World Economic Forum , Women’s Cancer is Getting Worse in Asia-Pacific–Here’s What to Do, 2023, https://www.weforum.org/stories/2023/10/womens-cancer-is-getting-worse-in-asia-pacific-heres-what-to-do/.

[10] National Cancer Registry of Pakistan , First Comprehensive Report of Cancer Statistics 2015–2019, Journal of College of Physicians and Surgeons Pakistan. (2021) 33, no. 6. 10.29271/jcpsp.2023.06.625 37300256

[11] Khan N. H. , Duan S. F. , Wu D. D. , and Ji X. Y. , Better Reporting and Awareness Campaigns Needed for Breast Cancer in Pakistani Women, 2021, Cancer Management and Research. 10.2147/CMAR.S270671 PMC7936924 33688255

[12] International Agency for Research on Cancer (IARC) , Global Cancer Observatory: Cancer Today, 2020, International Agency for Research on Cancer, https://gco.iarc.fr/today.

[13] World Health Organization , Breast Cancer Awareness Event, 2020, World Health Organization, https://www.emro.who.int/pak/pakistan-events/breast-cancer-awareness-event.html.

[14] Fairoosa P. and Witharana C. , Gene Mutations in Hereditary Breast Cancer-A Review, European Journal of Medical and Health Sciences. (2020) 2, no. 3, 10.24018/ejmed.2020.2.3.286.

[15] Breast Cancer Association Consortium , Breast Cancer Risk Genes—Association Analysis in More Than 113,000 Women, New England Journal of Medicine. (2021) 384, no. 5, 428–439, 10.1056/nejmoa1913948. 33471991 PMC7611105

[16] Dubsky P. , Jackisch C. , Im S. A. et al., *BRCA* Genetic Testing and Counseling in Breast Cancer: How Do We Meet Our Patients’ Needs?, NPJ Breast Cancer. (2024) 10, no. 1, 10.1038/s41523-024-00686-8. PMC11377442 39237557

[17] Kuchenbaecker K. B. , Hopper J. L. , Barnes D. R. et al., Risks of Breast, Ovarian, and Contralateral Breast Cancer for *BRCA1* and *BRCA2* Mutation Carriers, JAMA. (2017) 317, no. 23, 2402–2416, 10.1001/jama.2017.7112, 2-s2.0-85021167654. 28632866

[18] Rosen E. M. , Fan S. , Pestell R. G. , and Goldberg I. D. , *BRCA1* Gene in Breast Cancer, Journal of Cellular Physiology. (2003) 196, no. 1, 19–41, 10.1002/jcp.10257, 2-s2.0-0037946789. 12767038

[19] Balmana J. , Diez O. , Castiglione M. , and Esmo Guidelines Working Group , *BRCA* in Breast Cancer: ESMO Clinical Recommendations, Annals of Oncology. (2009) 20, 419–420, 10.1093/annonc/mdp116, 2-s2.0-66549093575. 19454451

[20] Antoniou A. , Pharoah P. D. , Narod S. et al., Average Risks of Breast and Ovarian Cancer Associated With *BRCA1* or *BRCA2* Mutations Detected in Case Series Unselected for Family History: A Combined Analysis of 22 Studies, The American Journal of Human Genetics. (2003) 72, no. 5, 1117–1130, 10.1086/375033, 2-s2.0-0038744296. 12677558 PMC1180265

[21] Jang E. R. and Lee J. S. , DNA Damage Response Mediated Through *BRCA1* , Cancer Research and Treatment. (2004) 36, no. 4, 214–221, 10.4143/crt.2004.36.4.214. 20368837 PMC2843892

[22] Petrucelli N. , Daly M. B. , and Feldman G. L. , Hereditary Breast and Ovarian Cancer Due to Mutations in *BRCA1* and *BRCA2* , Genetics in Medicine. (2010) 12, no. 5, 245–259, 10.1097/gim.0b013e3181d38f2f, 2-s2.0-77952541315. 20216074

[23] Hondow H. L. , Fox S. B. , Mitchell G. et al., A High-Throughput Protocol for Mutation Scanning of the *BRCA1* and *BRCA2* Genes, BMC Cancer. (2011) 11, 265–11, 10.1186/1471-2407-11-265, 2-s2.0-79959389584. 21702907 PMC3146935

[24] Takano E. A. , Mitchell G. , Fox S. B. , and Dobrovic A. , Rapid Detection of Carriers With *BRCA1* and *BRCA2* Mutations Using High Resolution Melting Analysis, BMC Cancer. (2008) 8, 59–7, 10.1186/1471-2407-8-59, 2-s2.0-40749117206. 18298804 PMC2266761

[25] Raponi M. , Smith L. D. , Silipo M. , Stuani C. , Buratti E. , and Baralle D. , *BRCA1* Exon 11 a Model of Long Exon Splicing Regulation, RNA Biology. (2014) 11, no. 4, 351–359, 10.4161/rna.28458, 2-s2.0-84901398341. 24658338 PMC4075520

[26] Deng C. X. , *BRCA1*: Cell Cycle Checkpoint, Genetic Instability, DNA Damage Response and Cancer Evolution, Nucleic Acids Research. (2006) 34, no. 5, 1416–1426, 10.1093/nar/gkl010, 2-s2.0-33645003172. 16522651 PMC1390683

[27] Sambrook J. and Russell D. W. , Detection of DNA in Agarose Gels, Molecular Cloning, A Laboratory Manual. (2001) 3rd edition, Cold Spring Harbor Laboratory Press, 5–14.

[28] Yates A. D. , Achuthan P. , Akanni W. et al., Ensembl 2020, Nucleic Acids Research. (2020) 48, no. D1, D682–D688, 10.1093/nar/gkz966. 31691826 PMC7145704

[29] Malik S. , Bibi A. , Farid R. , Khan S. , Awan J. , and Rehman A. U. , Consanguinity in Northwest Pakistan: Evidence of Temporal Decline, Journal of Biosocial Science. (2024) 56, no. 3, 445–458, 10.1017/s0021932024000026. 38314634

[30] Ullah M. , Rehman A. U. , Quinodoz M. et al., A Comprehensive Genetic Landscape of Inherited Retinal Diseases in a Large Pakistani Cohort, NPJ genomic medicine. (2025) 10, no. 1, 10.1038/s41525-025-00488-2. PMC11968986 40180963

[31] Ur Rehman A. , Peter V. G. , Quinodoz M. et al., Exploring the Genetic Landscape of Retinal Diseases in North-Western Pakistan Reveals a High Degree of Autozygosity and a Prevalent Founder Mutation in *ABCA4* , Genes. (December 2019) 11, no. 1, 10.3390/genes11010012. PMC7017091 31877759

[32] Ferla R. , Calo V. , Cascio S. et al., Founder Mutations in *BRCA1* and *BRCA2* Genes, Annals of Oncology. (2007) 18, vi93–vi98, 10.1093/annonc/mdm234, 2-s2.0-35748929114. 17591843

[33] Muhammad N. , Azeem A. , Arif S. et al., Prevalence of *BRCA1* and *BRCA2* Germline Variants in an Unselected Pancreatic Cancer Patient Cohort in Pakistan, Hereditary Cancer in Clinical Practice. (2023) 21, no. 1, 10.1186/s13053-023-00269-x. PMC10640758 37951914

[34] Rashid M. U. , Muhammad N. , Naeemi H. , Shehzad U. , and Hamann U. , Chasing the Origin of 23 Recurrent *BRCA1* Mutations in Pakistani Breast and Ovarian Cancer Patients, International Journal of Cancer. (2022) 151, no. 3, 402–411, 10.1002/ijc.34016. 35377489

[35] Ahmad H. , Ali A. , Ali R. et al., Preliminary Insights on the Mutational Spectrum of *BRCA1* and *BRCA2* Genes in Pakhtun Ethnicity Breast Cancer Patients From Khyber Pakhtunkhwa (KP), Pakistan, Neoplasia. (2024) 51, 10.1016/j.neo.2024.100989. PMC11026844 38537553

[36] Siddique A. , Fatima W. , and Shahid N. , Association of Common *BRCA1* Variants With Predisposition to Breast Tumors in Pakistan, Annals of Human Genetics. (2023) 87, no. 5, 222–231, 10.1111/ahg.12511. 37191028

[37] Schwartz M. , Ibadioune S. , Delhomelle H. et al., High Prevalence of Constitutional *BRCA1* Epimutation in Patients With Early-Onset Triple-Negative Breast Cancer, Clinical Epigenetics. (2025) 17, no. 1, 10.1186/s13148-025-01885-1. PMC12135219 40462235

[38] Muhammad N. , Azeem A. , Bakar M. A. et al., Contribution of Constitutional *BRCA1* Promoter Methylation to Early-Onset and Familial Breast Cancer Patients From Pakistan, Breast Cancer Research and Treatment. (2023) 202, no. 2, 377–387, 10.1007/s10549-023-07068-x. 37528266

