# Correction to “*BRCA1* Exon 11 Mutations in Breast Cancer: A Study From Pakistan”

**DOI:** 10.1155/genr/9873852

**Published:** 2026-03-12

M. Ali, A. Uddin, S. U. Ghafoor, and A. U. Rehman, “*BRCA1* Exon 11 Mutations in Breast Cancer: A Study From Pakistan,” *Genetics Research*, 2025, 5544418, https://doi.org/10.1155/genr/5544418.

In the article, the following affiliation for Murad Ali was erroneously omitted:

• Department of Internal Medicine and Medical Specialties (DIMI), University of Genoa, Genoa, Italy

This has been corrected in the published article.

We apologize for this error.

